# Primary Hypoparathyroidism Presenting as Idiopathic Intracranial Hypertension in a Patient With Barakat Syndrome

**DOI:** 10.7759/cureus.24521

**Published:** 2022-04-27

**Authors:** Hussam R Alkaissi, Mary A Banerji

**Affiliations:** 1 Internal Medicine, Kings County Hospital Center, Brooklyn, USA; 2 Internal Medicine, Veterans Affairs Medical Center, Brooklyn, USA; 3 Internal Medicine, State University of New York Downstate Medical Center, Brooklyn, USA; 4 Endocrinology, State University of New York Downstate Medical Center, Brooklyn, USA; 5 Endocrinology, Kings County Hospital Center, Brooklyn, USA

**Keywords:** genetic syndromes, persistent hypocalcemia, barakat syndrome, intracranial idiopathic hypertension, congenital deafness, primary hypoparathyroidism

## Abstract

Unlike hyperparathyroidism, hypoparathyroidism is rarely encountered in clinical practice. Usually, it results from surgical resection, an autoimmune phenomenon, or an infiltrative process. Under certain circumstances, one may encounter a genetic etiology of hypoparathyroidism, often combined with myriad other syndromic manifestations. We report a case of a young female with congenital deafness and subacute visual loss. Hypocalcemia and primary hypoparathyroidism were subsequently discovered, and the cause of the vision loss was diagnosed as idiopathic intracranial hypertension, likely secondary to severe primary hypoparathyroidism. The patient was also found to have small bilateral kidneys, with tubular loss of magnesium and calcium, yet with a normal glomerular filtration rate. The constellation of congenital deafness, hypoparathyroidism, and renal dysfunction suggests Barakat syndrome, one of the less common causes of syndromic primary hypoparathyroidism.

## Introduction

Hypoparathyroidism is rarely encountered on clinical grounds, and it is often seen after surgical resection, an autoimmune phenomenon in patients with other autoimmune diseases or infiltrative diseases [1]. Rarely a disease of genetic etiology manifests in adulthood. Hypoparathyroidism's genetic causes range from isolated hypoparathyroidism to a syndromic constellation of manifestations such as DiGeorge syndrome, Sanjad-Sakati syndrome, and Barakat syndrome [2]. Here, we report hypoparathyroidism, manifesting as idiopathic intracranial hypertension (IIH) with related visual loss, in an adult patient with congenital deafness and renal abnormalities suggestive of Barakat syndrome.

## Case presentation

A 28-year-old female with congenital sensory neural hearing loss presented with subacute gradual visual loss over two months. The history was obtained using sign language. The patient states that she can see some light and shapes, but her vision has declined over the past two months, and she cannot perform her daily activities. She had no eye pain, redness, lacrimation, or foreign body sensation. She also complained of headache, worse in the morning, with no nausea or vomiting. The patient also complained of long-standing increased urinary frequency and nocturia (10-15 times a day), with no dysuria, or obstructive urinary symptoms. No fever or weight loss. No prior surgeries. No history of renal disease, deafness, or hypoparathyroidism in the family.

On examination, vitals signs were normal. An ophthalmological exam revealed bilateral papilledema, normal extraocular movement, and light reflex, but Humfrey's visual acuity was difficult to assess given the patient's limited communication. Initial blood work showed hypocalcemia (calcium down to 4.7 mg/dL) with low parathyroid hormone levels (PTH) of 18.2 pg/mL, consistent with primary hypoparathyroidism (Table 1). She was started on calcium carbonate 2500 mg orally every six hours and magnesium oxide 400 mg orally once daily.

**Table 1:** Laboratory data on admission and a week later after the patient was treated with calcium and vitamin D supplementation. eGFR: estimated glomerular filtration rate; 25(OH) vitamin D: 25-hydroxycholecalciferol

Values	On admission	Day 7	Reference range
Sodium (mmol/L)	142	141	135-145
Potassium (mmol/L)	3.6	3.7	3.5-5.1
Chloride (mmol/L)	103	103	98-107
Carbon dioxide (mmol/L)	20	25	21-31
Urea nitrogen (mg/dL)	8	11	7-25
Creatinine (mg/dL)	0.85	0.74	0.6-1.2
eGFR (ml/min/1.73 m^2^)	103	110	>60
Calcium (mg/dL)	4.7	8	8.2-10
Ionized calcium (mmol/L)	0.6	NA	1.16-1.32
Magnesium (mg/dL)	1.27	2	1.3-2.1
Phosphate (mg/dL)	4.3	NA	2.5-4.5
Albumin (g/dl)	3.7	NA	3.5-5.7
Parathyroid hormone (pg/mL)	18.2	17.7	15-65
25(OH) Vitamin D (ng/mL)	10.1	NA	>30

**Figure 1:**
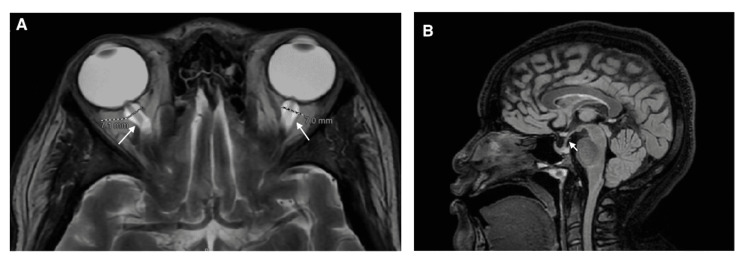
Brain MRI showing signs of an increased intracranial pressure without space-occupying lesions. (a) Axial T2-weighted imaging (T2WI) showing optic nerve sheath edema, evidenced by an increase in optic nerve sheath diameter (OSND) of > 6 mm bilaterally (normal OSND between 4.8 and 6 mm). (b) Sagittal fluid-attenuated inversion recovery (FLAIR) sequence showing empty sella turcica with infundibulum sign.

CT scan of the head revealed partially empty sella turcica. MRI confirmed complete empty sella (>50% of the sella full with cerebrospinal fluid {CSF}), with a positive infundibulum sign. Optic nerve sheaths were edematous on T2-weighted imaging, with dilated caliber (right optic nerve {ON} diameter was 7 mm, left ON diameter 7.1 mm, normal <6 mm), with no space-occupying lesion, findings suggestive of idiopathic intracranial hypertension (IIH) (Figure 1). Thyroid-stimulating hormone and other pituitary hormones were within the normal range. Urinalysis was unremarkable apart from asymptomatic bacteriuria. No proteinuria on urinalysis.

A lumbar puncture was performed, showing an elevated opening pressure of 31 cm H_2_O (normal pressure from 10-20 cm H_2_O). Therefore, a diagnosis of IIH was confirmed, and the patient was started on acetazolamide 750 mg twice daily. Given the patient's young age, a constellation of deafness and hypoparathyroidism, and history of polyuria, further renal studies were done. Renal ultrasound show bilateral small kidneys (right kidney length was 8 cm, left kidney length was 9, normal adult kidney length is between 10 and 12 cm) with preserved fetal lobulation-otherwise, normal cortico-medullary differentiation, no stones or hydronephrosis, and no evidence of nephrocalcinosis.

Urine electrolytes were measured on a spot urine sample and 24-hour urine collection (Table 2). The 24-hour urine volume was 3 liter, with calcium levels of 463 mg/day (reference range: < 200 mg/day) [3]. She was also found to have hypermagnesuria, with fractional excretion of magnesium (FeMg) of 9% (reference range: 2-4%) [4]. The rest of the urine electrolytes were unremarkable. Of note, calcium clearance doubled from 1% to 2% after she was started on the calcium gluconate and vitamin D, indicating hypercalciuria. 

**Table 2:** Urine electrolytes analysis from spot urine samples collected on admission, and a week later after the patient was started on calcium and vitamin D supplementation. Twenty-four-hour urine collection confirmed hypercalciuria. FeCa: fractional excretion of calcium; FeMg: fractional excretion of magnesium

Values	On admission	Day 7	Reference range	Author, year
Spot urine creatinine (mg/dL)	33.24	36.7	28-217	-
Spot urine calcium (mg/dL)	2.2	8	-	-
Spot FeCa (%)	1	2	1-2	Black et al., 2013 [3]
Spot urine magnesium (mg/dL)	NA	8.3	-	-
Spot FeMg (%)	NA	9	2-4	Elisaf et al., 1997 [4]
24-hour urine calcium (mg)	-	463	100-300	-
24-hour urine volume (L)	-	3	-	-

The patient developed an episode of acute kidney injury, with a reduction of estimated glomerular filtration rate (eGFR) from 100 down to 25-30 ml/min. A repeated spot of urine electrolytes showed a fractional excretion of sodium (FeNa) of 10%. No apparent cause was identified, but the fact that she developed hypercalciuria after starting calcium supplement led us to believe that the calcium loss might be related to the kidney injury. She was started on IV fluids, calcium supplements were reduced, and the patient's kidney function recovered over five days. Calcium levels were kept at a lower level of around 7 mg/dL. She was discharged on acetazolamide and followed closely with neurology with gradual improvement in vision.

## Discussion

In 1977, Barakat et al. described family members with congenital deafness, hypoparathyroidism, and severe renal disease (steroid-resistant nephrosis). Further examination of the family showed a milder disease phenotype with isolated deafness in other members of the same family. The name Barakat syndrome was applied, or HDR, "H" for hypoparathyroidism, "D" for deafness, and "R" for renal disease [5]. Bilous et al. reported a similar syndrome in another family in 1992 and, Van Esch et al. in 2000, were able to attribute the syndrome to haploinsufficiency in the gene of the transcription factor GATA3 [6,7].

Barakat syndrome is an autosomal dominant disease with incomplete penetrance. One hundred eighty cases have been recently reviewed by Barakat et al. Hearing loss to complete deafness was the most common component of the syndrome, present in over 96% of the patients. The second most common manifestation was hypoparathyroidism, present in 93% of the patient, either as low PTH levels or inappropriately normal in patients with hypocalcemia. Renal involvement is less common, affecting 72.2%, ranging from structural defects such as small kidneys to functional defects, such as proteinuria and tubular defect. About 90% of the patient had a loss of function mutation of GATA3, a zinc finger that function as a transcription factor for parathyroid development and is also known as a master regulator of T-helper type 2 (Th2) [5].

We present a patient with congenital sensory neural hearing loss who presented with visual loss. Hypocalcemia was found incidentally, as there were no apparent signs and symptoms such as tetany or seizures. This led to the diagnosis of primary hypoparathyroidism, given the inappropriately low normal PTH levels on two occasions (Table 1). This, in turn, with papilledema on the examination, was a clue that the patient may have idiopathic intracranial hypertension (IIH) secondary to her severe hypocalcemia and hypoparathyroidism. The association between hypoparathyroidism and IIH is anecdotal yet frequently reported and is thought to be secondary to the calcium-dependent function of the arachnoid villi to absorb CSF [8-14]. Adding hypoparathyroidism to the picture led us to investigate the possibility of Barakat syndrome. We found that the patient has small kidneys, with hypercalciuria and hypermagnesuria, yet with a normal estimated glomerular filtration rate [3,4,15].

Important differential diagnoses had to be excluded first to establish the diagnosis of Barakat syndrome. The presence of hypermagnesuria can indicate familial hypocalcemia, hypomagnesemia, and nephrocalcinosis (FHHN). Patients FHHN have defects in claudin 16 and 19 that form heterodimers between the tubular cells of the thick ascending limb of Henle's loop (TAL) and distal convoluted tubule (DCT) cells that aid in calcium and magnesium absorption. Those patients have secondary hyperparathyroidism rather than hypoparathyroidism and almost invariably have nephrocalcinosis. Our patient has no nephrocalcinosis on ultrasound and hypoparathyroidism, making this diagnosis unlikely. Hypomagnesemia can lead to hypoparathyroidism and hypocalcemia due to its effect on calcium-sensing receptor (CaSR) signaling, but this is seen in much lower magnesium levels. Our patient was able to maintain normal magnesium levels despite her significant renal loss of magnesium, likely due to increasing enteral absorption. Similarly, in isolated dominant hypomagnesemia (IDH), despite having similarly high FeMg, these patients have more severe hypomagnesemia with seizures and myriad neurological manifestations such as ataxia, muscle weakness, and myokymia and do not have deafness or blindness [16].

Certain renal tubulopathies, such as subsets of Barter syndrome, can cause deafness, but that is also unlikely in our case due to the absence of salt wasting and contraction alkalosis [15]. Mitochondrial diseases can manifest as deafness and hypoparathyroidism, but these patients often have myopathy and elevated lactate levels [1]. However, our patient's lactate levels were normal, and she had no other stigmata of mitochondrial diseases (e.g., no retinitis pigmentosa on fundoscopic eye examination, no cardiac conduction defects).

Finally, an important cause of hypoparathyroidism and hypercalciuria is defects in calcium-sensing receptor (CaSR), known as autosomal dominant hypoparathyroidism (ADH). Most commonly, genetic gain-of-function, known as autosomal dominant hypocalcemia (ADH), leads to over-sensitive CaSR, suppressing the PTH release and renal calcium reabsorption at much lower levels of calcium, with a PTH-calcium inverse sigmoid curve shifted to the left [1,15]. Some rare acquired causes can be seen in hypomagnesemia (mentioned above) and stimulating autoantibodies [16]. Pearce et al. showed that calcium supplementation to patients with autosomal dominant hypocalcemia leads to suppression of PTH levels from low normal to undetectable levels due to heightened CaSR sensitivity yet functional feedback loop. Our patient’s calcium went up from 4.7 to 8.4 mg/dL after calcium supplementation yet with no changes in PTH levels (18.2-17.7 pg/dL). This lack of feedback inhibition makes autosomal dominant hypoparathyroidism an unlikely cause of our patient's presentation [15,17,18].

It is likely that the renal magnesium and calcium loss, with small volume, and polyuria, are primary renal manifestations of Barakat syndrome, as it would also explain the deafness and the hypoparathyroidism. The delayed manifestation of hypoparathyroidism is in concordance with the patients reviewed by Barakat et al. As deafness is usually the earliest manifestation of the syndrome, hypoparathyroidism, and renal disease can follow years to decades later. The main weakness in our case is that genetic testing was unavailable, yet, it is not necessary to make the diagnosis. The syndrome is defined as the HDR triad or any two of its components with strong family history. Up to 10% of reported cases have no GATA3 mutations. This can be explained by defects elsewhere in the genome, such as in other transcription factors involved in parathyroid development, such as GCM2 [2].

## Conclusions

We presented an unusual case of primary hypoparathyroidism that did not exhibit the telltale symptoms and signs of hypocalcemia but instead presented as visual loss secondary to idiopathic intracranial hypertension. We would like to draw attention to the possibility of syndromic and genetic causes of hypoparathyroidism presenting in adulthood. Knowledge of the possibility of renal involvement in such patients with Barakat syndrome helped identify early signs of renal abnormalities such as hypercalciuria and hypermagnesuria.
